# Lithium chloride induces mesenchymal-to-epithelial reverting transition in primary colon cancer cell cultures

**DOI:** 10.3892/ijo.2015.2911

**Published:** 2015-03-02

**Authors:** VALERIA COSTABILE, FRANCESCA DURATURO, PAOLO DELRIO, DANIELA REGA, UGO PACE, RAFFAELLA LICCARDO, GIOVANNI BATTISTA ROSSI, RITA GENESIO, LUCIO NITSCH, PAOLA IZZO, MARINA DE ROSA

**Affiliations:** 1 Department of Molecular Medicine and Medical Biotechnology, University of Naples Federico II, I-80131 Naples, Italy; 2 Colorectal Surgical Oncology, Istituto Nazionale per lo Studio e la Cura dei Tumori, ‘Fondazione Giovanni Pascale’ IRCCS, I-80131 Naples, Italy; 3 Endoscopy Unit, Istituto Nazionale per lo Studio e la Cura dei Tumori, ‘Fondazione Giovanni Pascale’ IRCCS, I-80131 Naples, Italy; 4 CEINGE Biotecnologie Avanzate, I-80145 Naples, Italy

**Keywords:** colorectal cancer, primary colon cancer cell cultures, epithelial-to-mesenchymal transition, mesenchymal-to-epithelial transition, stem cell-like phenotype, GSK3β inhibition

## Abstract

Epithelial-to-mesenchymal transition (EMT) confers stem cell-like phenotype and more motile properties to carcinoma cells. During EMT, the expression of E-cadherin decreases, resulting in loss of cell-cell adhesion and increased migration. Expression of Twist1 and other pleiotropic transcription factors, such as Snail, is known to activate EMT. We established primary colon cancer cell cultures from samples of operated patients and validated cultures by cytogenetic and molecular biology approaches. Western blot assay, quantitative real-time PCR and immunofluorescence were performed to investigate the expression of E-cadherin, vimentin, β-catenin, cytokeratin-20 and -18, Twist1, Snail, CD44, cyclooxygenase-2 (COX2), Sox2, Oct4 and Nanog. Moreover, cell differentiation was induced by incubation with LiCl-containing medium for 10 days. We observed that these primary colorectal cancer (CRC) cells lost expression of the E-cadherin epithelial marker, which was instead expressed in cancer and normal colon mucosa of the same patient, while overexpressed vimentin (mesenchymal marker), Twist1, Snail (EMT markers) and COX2. Cytokeratin-18 was expressed both in tissues and cell cultures. Expression of stem cell markers, such as CD44, Oct4 and Nanog, were also observed. Following differentiation with the glycogen synthase kinase 3β (GSK3β) inhibitor LiCl, the cells began to express E-cadherin and, at once, Twist1 and Snail expression was strongly downregulated, suggesting a MET-reverting process. In conclusion, we established primary colon mesenchymal cancer cell cultures expressing mesenchymal and epithelial biomarkers together with high level of EMT transcription factors. We propose that they could represent a good model for studying EMT and its reverting mechanism, the mesenchymal-to-epithelial transition (MET). Our observation indicates that LiCl, a GSK3β inhibitor, induces MET *in vitro*, suggesting that LiCl and GSK3β could represent, respectively, interesting drug, and target for CRC therapy.

## Introduction

Colorectal cancer (CRC) is the second cause of cancer deaths in Europe and the third in the United States, with >1 million new cases estimated every year. Death occurs because of metastasis to other organs (1).

Epithelial cells from gastrointestinal trait acquire sequential genetic and epigenetic mutations in specific oncogene and/or tumor suppressor genes, during colorectal adenocarcinoma development, conferring them a selective advantage on proliferation and self-renewal (2). Thus, normal epithelium becomes hyperproliferative mucosa and subsequently gives rise to a benign adenoma that progressively evolves into carcinoma and, subsequently, generates metastasis (3–5).

Loss of genomic integrity facilitates the accumulation of multiple mutations during the development of CRC. Chromosomal instability (CIN), microsatellite instability (MIN), aberrant DNA methylation and DNA repair defects are all mechanisms involved in colorectal epithelial cell transformation (6).

At molecular level CRCs are a very heterogeneous group of diseases showing several alterated molecular signaling pathways, such as Wnt/APC/β-catenin, PI3K/Akt/glycogen synthase kinase 3β (GSK3β), TGFβ/SMAD, NF-κB or mismatch repair genes (MMR). These alterations confer individual susceptibility to cancer, and are responsible for responsiveness or resistance to antitumor agents (7).

Wnt/β-catenin pathway is the most frequently dysfunctional signaling in sporadic CRC. When Wnt ligand, a secreted glycoprotein, binds to its frizzled receptors, the multifunctional kinase GSK3β is inactivated and β-catenin, that acts both as E-cadherin cell-cell adhesion protein and as a transcriptional activator, is stabilized, accumulated in the cytoplasm and finally translocates into nucleus. Here it interacts with members of the lymphoid enhancer factor (LEF)/T-cell factor (TCF) and activates specific target genes. In the absence of Wnt signal, casein kinase 1 (CK1) and the APC/Axin/GSK3β complex, target β-catenin for ubiquitination and proteasomal degradation by its phosphorylation (8).

GSK3β is a multifunctional serine/threonine kinase and an important regulator of cell survival that may act as anti- or pro-apoptotic, in a cell-specific manner. Its activity is regulated by site-specific phosphorylation. Full activity of GSK3β generally requires phosphorylation at tyrosine 216 (Tyr216), whereas phosphorylation at serine 9 (Ser9) inhibits its activity. It has been demonstrated that activation of PI3K and PKC inhibits GSK3β activity by stimulating its Ser9 phosphorylation (9).

GSK3β also regulates NF-κB activity, a transcription factor that plays a role in many physiological and pathophysiological processes, including immune responses, inflammation, cell proliferation, survival and differentiation. In colon and pancreatic cancer cells, NF-κB is positively regulated by GSK3β and its activation confers a selective growth advantage on these cells, so acting as a tumor promoter. The molecular mechanisms underlying GSK3β/NF-κB interaction remain to be further investigated (10,11). More than 100 proteins, playing a role in a wide spectrum of cellular processes, are substrates of GSK3β, among which, β-catenin and NF-κB inhibitor IκB are the most well-known. Genes upregulated by β-catenin/TCF/LEF and/or NF-κB include proto-oncogenes, such as *c-Myc* and *cyclin-D1*, and genes regulating cell invasion/migration, such as *Snail*, *CD44* and *MMP-7* (12).

Recently, it has been suggested that epithelial-to-mesenchymal transition (EMT) could be a common biological mechanism that could represent a good target for therapeutic intervention. EMT consists in an essential phenotypic conversion of epithelial cells into cells with mesenchymal phenotype. It is a reversible process that often occurs during embryonic development and tissue remodeling and also plays a critical role in early events occurring in invasion and metastasis of many types of cancer, including CRC (13). EMT regulation is orchestrated by a group of transcription factors, including Snail, Slug, ZEB1 and Twist, but tumor microenvironment also plays a part into phenotypic conversion through different signals, such as TGFβ, EGF, Wnt and Notch (14,16).

During EMT epithelial cells lose their E-cadherin expression, that specifically guarantees the epithelial phenotype, destroy their intercellular adhesion, acquire mesenchymal characteristics and increase migratory and invasive properties. Furthermore, the EMT program induces stem cell-specific gene expression, thus promoting self-renewal capability (14–16).

One of the main problems in cancer treatment is drug resistance, responsible for relapses in many tumors and the failure of medical treatments in metastatic disease. Probably, both chemotherapy and radiation therapy too often miss the opportunity to kill a part of a tumor cell subpopulation, such as CSC and CSC-like cells.

We aimed to realize a tissue biobank from patients affected by CRC and to establish primary cell cultures with the main purpose of studying EMT and its reverting mechanism, the mesenchymal-to-epithelial transition (MET) in CRC, *in vitro*. We also investigated GSK3β inhibition as therapeutic target of CRC.

## Materials and methods

### Patients

Blood samples, normal colorectal mucosa and CRC tissues were obtained from patients with sporadic colon cancer operated at the Istituto Nazionale dei Tumori in Naples (Italy) and tissues were frozen in liquid nitrogen. Primary cell cultures were also established from some of these CRC patients.

Samples from all subjects who participated in the study were collected after being granted authorization from the Comitato Etico per le Attività Biomediche ‘Carlo Romano’ of the University of Naples Federico II, with protocol no. 120/10. Such authorization is given only once the study has received ethics approval, and the participants informed and written consent has been obtained.

### Cell cultures

Samples of CRC from CRC patients were washed overnight at 4°C in PBS containing 300 U/ml penicillin, 300 μg/ml streptomycin, and 2.5 μg/ml amphotericin B (all from Gibco-BRL, Karlsruhe, Germany), finely minced with scissors (tissue pieces of ~30 mm^3^) and digested in 2 ml 0.1% collagenase II (Boehringer Mannheim, Mannheim, Germany) in DMEM/FBS-10% for 1 h at 37°C, 5% CO_2_. The cell suspension was then collected by centrifugation, washed twice with serum-free DMEM medium, and subsequently cultured in DMEM/F12-10% FBS medium (1:1), 100 U/ml penicillin, 100 μg/ml streptomycin, and 2.5 μg/ml amphotericin B (all from Gibco-BRL). CRC cells were selected by differential sedimentation, cultured on plates, and incubated with LiCl (30 mmol/l) for 1 and 24 h as well as 10 days. For *in vitro* differentiation, DMEM/F12-5% FBS medium 30 mM LiCl with was used. These primary colon cancer cells were then cultured as spheres in serum-free stem cell medium and low-adhesion plates, as described by Kreso and O’Brien (17), for ~60 days, disgregated six times every 10 days.

### Cytogenetic analysis

Metaphase chromosome analysis was performed on cell cultures from CRC patients, using high resolution G-banding (550 bands) according to standard procedures. Multicolor-FISH (M-FISH) was carried out using MetaSystems’ 24XCyte color kit (MetaSystems GmbH, Altlussheim, Germany).

FISH analysis was performed using whole chromosome painting (WCP) probes for chromosomes 20 and 22 and locus-specific DiGeorge probe mixture (MetaSystems GmbH) that contains a SpectrumOrange probe located at 22q11.2 and a SpectrumGreen LSI probe that maps at 22q13.3 region and subtelomeric probes for the p (green) and q (red) arms of chromosomes 20.

Multicolor chromosome banding (MCB) was performed using the multicolor banding DNA probe kit based on micro-dissection derived region-specific libraries for chromosome 22 (MetaSystems GmbH) according to standard protocols (18).

FISH experiments were performed on metaphase spreads and fluorescent images were analysed using a fluorescence microscope (Axio Imager.Z1 mot; Carl Zeiss Microscopy, LLC, Thornwood, NY, USA) with ISIS software imaging system (MetaSystems GmbH) for image capturing and processing.

### RER assay

The MSI status was confirmed with a fluorescent multiplex system including six mononucleotide repeats (BAT-25, BAT-26, BAT-40, NR21, NR24, and TGFβRII) and four dinucleotide repeats (D2S123, D5S346, D17S250, and D18S58) as described earlier (19), using the CC-MSI kit (AB Analitica s.r.l., Padova, Italy), according to the manufacturer’s instructions. PCR products were analysed by capillary electrophoresis analysis using an ABI Prism 3130 Genetic Analyzer (Applied Biosystems, Inc., Foster City, CA, USA).

### RT-PCR analysis

Total RNA was extracted from colorectal tissues of CRC patients, using QIAzol Reagent (Qiagen, Hilden, Germany), after homogenization and cDNA was synthesized with 1 μg of total RNA, 500 ng of random hexamers, and 1 μl SuperScript III Reverse Transcriptase (Invitrogen Life Technologies, Carlsbad, CA, USA), in the presence of 4 μl 5X RT buffer, 1 μl DTT (0.1 M) and 1 mM dNTPs, after DNase incubation (Invitrogen Life Technologies). The reaction was run for 50 min at 42°C in a 20 μl reaction volume, heated to 70°C for 15 min and quickly chilled on ice. One microliter of the cDNA was amplified by RT-PCR for CTK20 and 18 as well as E-cadherin messengers, using primer pairs shown in Table I.

All oligonucleotides were obtained with Primer-BLAST Software (http://www.ncbi.nlm.nih.gov/tools/primer-blast/).

### Real-time PCR quantification analysis

Real-time PCR quantification analysis was performed for Twist1, Snail and cyclooxygenase-2 (COX2) messengers using 0.5 μl of the cDNA and primer pairs shown in Table I.

Relative expression was calculated with the comparative Ct method and normalized against the Ct of glucuronidase (GUS) mRNA. The quantitative real-time assays were performed using the Bio-Rad iCycler iQ Real-Time PCR Detection System (Bio-Rad Laboratories, Inc., Hercules, CA, USA) as previously described (20,21), using healthy mucosa (HM) as a calibrator to measure the relative expression.

### Western blot assay

Total proteins were extracted from HM of colon and colon tumor tissue (TT) of affected patients and from primary colon cancer cultures, using QIAzol Reagent (Qiagen) following the manufacturer’s instructions. Concentrations were determined by using a protein assay kit adopting bovine serum albumin standards, according to the manufacturer’s instructions (Bio-Rad Laboratories, Inc.). A total of 30 μg of proteins were separated by SDS-polyacrylamide gel electrophoresis and blots were prepared on a Amersham Hybond-ECL nitrocellulose membrane (Amersham Pharmacia Biotech, Inc./GE Healthcare Bio-Sciences Corp., Piscataway, NJ, USA). The primary antibody against β-catenin, E-cadherin (monoclonal, rabbit anti-human; no. 3195), CD44 (monoclonal, mouse anti-human; no. 5640), Snail (monoclonal, rabbit anti-human; no. 3879) and vimentin (monoclonal, rabbit anti-human; no. 5741) was from Cell Signaling Technology, Inc. (Beverly, MA, USA). The antibody against actin (polyclonal, rabbit anti-human; no. sc-1615) was from Santa Cruz Biotechnology, Inc. (Santa Cruz, CA, USA). The membrane was probed with a secondary antibody against peroxidase-conjugated rabbit or goat immunoglobulin G, and immunoreactive bands were detected using the enhanced chemiluminescence Immobilon Western HRP Substrate (Millipore, Billerica, MA, USA).

### Immunofluorescence

The primary cancer cells were seeded and grown on glass coverslips 24 h prior to the experiments. After fixation with 4% paraformaldehyde (PFA)-PBS for 10 min, cells were permeabilized in 0.1% Triton X-100 in PBS and sequentially blocked with 10% bovine serum albumin for 45 min. Following the overnight incubation with primary antibody specific for cytokeratin (pan) (no. 18-0059, 1:100 dilution; Zymed Laboratories, Inc., San Francisco, CA, USA), E-cadherin (no. 3195, 1:100 dilution), Nanog (polyclonal, rabbit anti-human, no. 3580, 1:100 dilution), Sox2 (monoclonal, mouse anti-human, no. 4900, 1:100 dilution), Oct4 (monoclonal, rabbit anti-human, no. 2840, 1:100 dilution) and Snail (no. 3879, 1:100 dilution), and/or 2 h incubation with primary antibody specific for vimentin (no. 5741, 1:200 dilution) and CD44 (no. 5640, 1:800 dilution) (all from Cell Signaling Technology, Inc.). Cells were further incubated with appropriate secondary antibodies and DAPI for nuclear labeling. The negative controls without primary antibodies were also included, while no obvious staining was observed. Immunofluorescence was visualized under a fluorescence microscope and image-captured.

## Results

### Stabilisation of primary colon cancer cell cultures and tumor status validation

**Figure 1:**
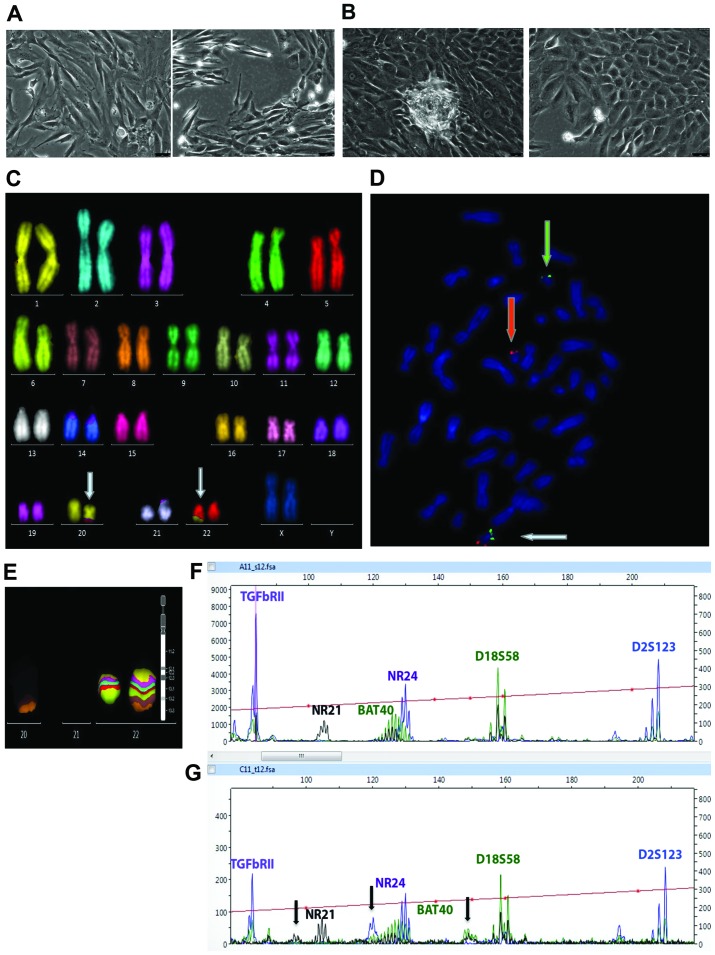
Molecular biology and cytogenetic validation of primary colon cultures. (A) T88 and (B) T93 cell culture images in a bright field with ×20 magnification (scale bar, 50 μm). (C–E) Karyotype analysis of T93 cell cultures; (C) multicolor-FISH (M-FISH) showing a reciprocal translocation between chromosomes 20 and 22; (E) high resolution multicolor chromosome banding (MCB) image of the normal chromosome 22 compared to the rearranged chromosome 22; (D) FISH with subtelomeric probes for the p (green) and q (red) arms of chromosome 20. (F and G) Microsatellite instability (MIN) assay of T88 cell culture performed on DNA from (F) peripheral blood cells and (G) tumor tissue (TT). Black arrows indicate altered microsatellites.

We realized a tissue biobank by sampling pairs of healthy colorectal mucosa and its matched CRC tissues from patients with sporadic and hereditary CRC, frozen in liquid nitrogen. From the same CRC tissues we established primary CRC cell cultures. For molecular investigation, we chose two different cultures: one mesenchymal-like (T88) (Fig. 1A) and the other more epithelial-like (T93) (Fig. 1B). According to TNM staging, classification of these two tumors was T3N1 and T4N2, for tumor 88 and 93, respectively. First of all, we validated the cancer status of our cultures using molecular biology and cytogenetic tecniques.

Karyotyping was performed on both cell cultures and cytogenetic analyses were interpreted at a resolution level of 550 bands. Only the T93 cells showed a female karyotype with a reciprocal translocation between the q arm of chromosome 20 and the q arm of chromosome 22, but no numerical aberrations were found (Fig. 1C). The high resolution MCB image of the normal chromosome 22 compared to the rearranged chromosome 22 shows that the 22q13.2qter region of chromosome 22 was translocated to chromosome 20, while the region of chromosome 20 translocated includes the region 20q13 to 20qter (Fig. 1E). FISH with subtelomeric probes for the p (green) and q (red) arms of chromosomes 20 shows that region 20q13 was translocated into chromosome 22 (Fig. 1D).

The complete chromosomal characterisation according to ISCN 2013 (22) was as follows: 46,XX,t(20;22)(q13;q13)(20pter→20q13.2::22q13.2→22qter:22pter→22q13.1::20q13.3→20qter).

MSI analysis was performed on DNA extracted from TT and pheripheral blood sample of patient no. 88, resulted negative for CIN phenotype, who was found to have an MSI-high (MSI-H) status, with instability at mononucleotide markers NR21, BAT-40 and NR24 (Fig. 1F and G).

### Cell cultures express mesenchymal and epithelial markers, EMT transcription factors and stemness markers

We have characterized T88 and T93 primary colon cancer cell cultures for epithelial and mesenchymal markers, by using RT-PCR, real-time PCR, western blot and immunofluorescent analysis.

**Figure 2:**
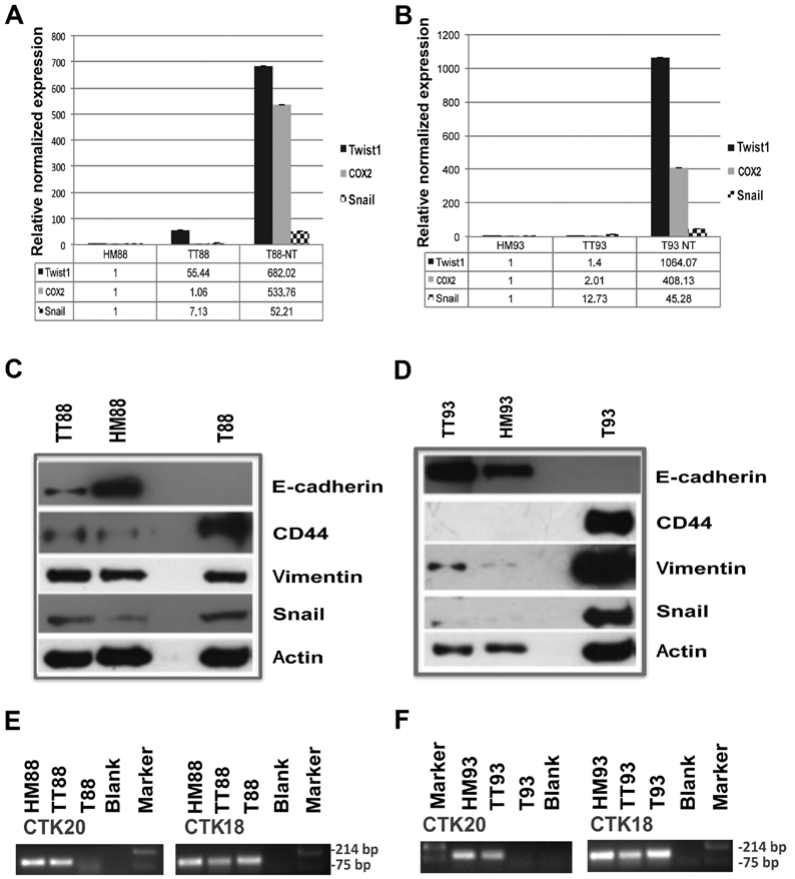
Protein and RNA expression analysis of T88 and T93 primary cell cultures. (A–B) Real-time RT-PCR analysis of Twist1, Snail and cyclooxygenase-2 (COX2), performed on healthy mucosa (HM), tumor tissue (TT), untreated tumor cell cultures (T) from patient (A) no. 88 and (B) no. 93. (C–D) Western blot assay of E-cadherin, CD44, vimentin, Snail and actin performed with 30 μg of proteins extracted from HM, TT and untreated T from patient (C) no. 88 and (D) no. 93. (E–F) RT-PCR analysis of cytokeratin 20 and 18 performed on cDNA from HM, TT and untreated T from patient (E) no. 88 and (F) no. 93.

As shown in Fig. 2A and B, Twist1, Snail and COX2 messengers were mainly upregulated in both cell cultures when investigated by real-time RT-PCR. GUS mRNA was used as calibrator in all real-time quantification assays and each HM of colon was adopted as target sample for relative quantification.

Furthermore, E-cadherin protein expression was downregulated in T88 and upregulated in T93 TTs, while completely absent in cell cultures. On the other hand, vimentin and Snail were upregulated in TTs, compared to normal colon mucosa of each patient but highly expressed in cell cultures, when analyzed by western blot assay. CD44, instead, was upregulated in both cell cultures, furthermore it was upregulated in TT from patient no. 88 but it gave no hybridization signal in tissues from patient no. 93, in our experimental conditions (Fig. 2C and D).

Moreover, HM of colon, colon cancer tissues and cell cultures expressed cytokeratin 18, whereas only healthy and cancer mucosa expressed cytokeratin 20, when analyzed by RT-PCR (Fig. 2E and F).

**Figure 3:**
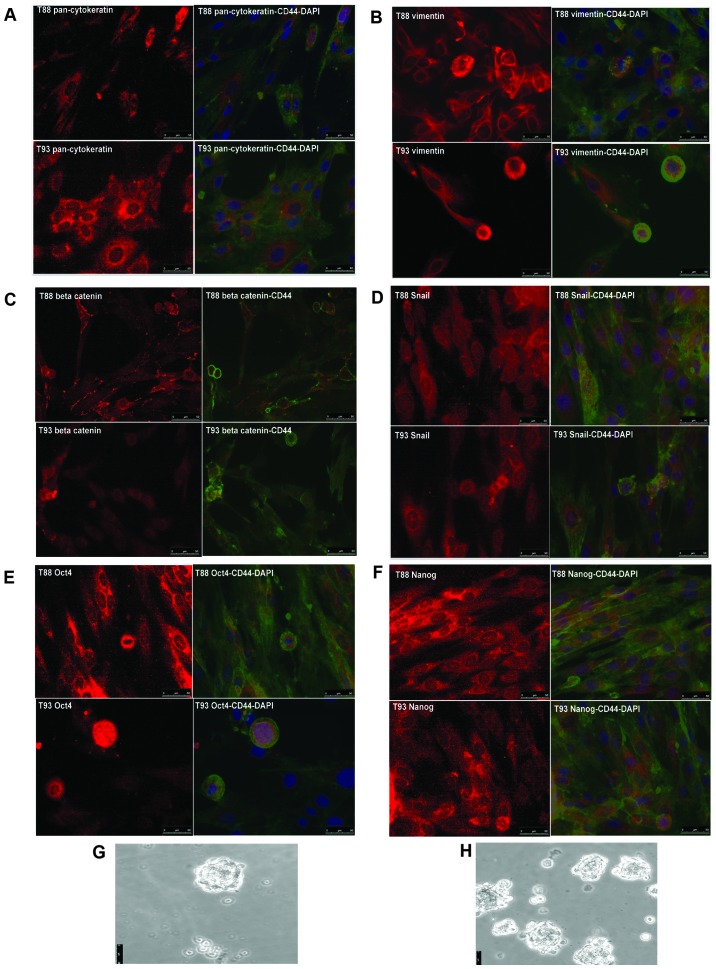
T88 and T93 primary cell cultures express mesenchymal, epithelial-to-mesenchymal transition (EMT) and stem cell characteristic. Immunofluorescence analysis performed on T88 and T93 cells of (A) cytokeratin (pan)/CD44, (B) vimentin/CD44, (C) β-catenin/CD44, (D) Snail/CD44, (E) Oct4/CD44, (F) Nanog/CD44 and (G and H) colon cancer spheres grown from T88 and T93 cells. Images in bright field. Magnification, ×20 (scale bar, 50 μm).

To clarify if all the cells of these cultures expressed mesenchymal markers, EMT-TF and epithelial markers or, alternatively, were a heterogeneous cell population, we performed an immunofluorescence assay using CD44, Snail, vimentin, pan-cytokeratin, Oct4, Nanog and Sox2 antibody. As shown in Fig. 3, all the cells were positive for all the analyzed proteins except for Sox2 protein, whose antibody did not give any signal in our experimental condition (data not shown). CD44 was mainly localized at membrane level (Fig. 3A–F), while pan-cytokeratine and vimentin were localized at the cytoskeleton level (Fig. 3A and B) and, as expected, β-catenin was localized at level of membrane, cytoplasm and nucleus (Fig. 3C). Snail, Oct4 and Nanog showed a turnover between nucleus and cytoplasm (Fig. 3D–F). Furthermore, Oct4 and Nanog were mostly expressed at the level of cancer cell spheres and clones (data not shown). They were also able to grow as a sphere in a conditioned medium without serum and in low adhesion condition (Fig. 3G and H)

### LiCl incubation of primary colon cancer cell culture induces MET and differentiation in vitro

**Figure 4:**
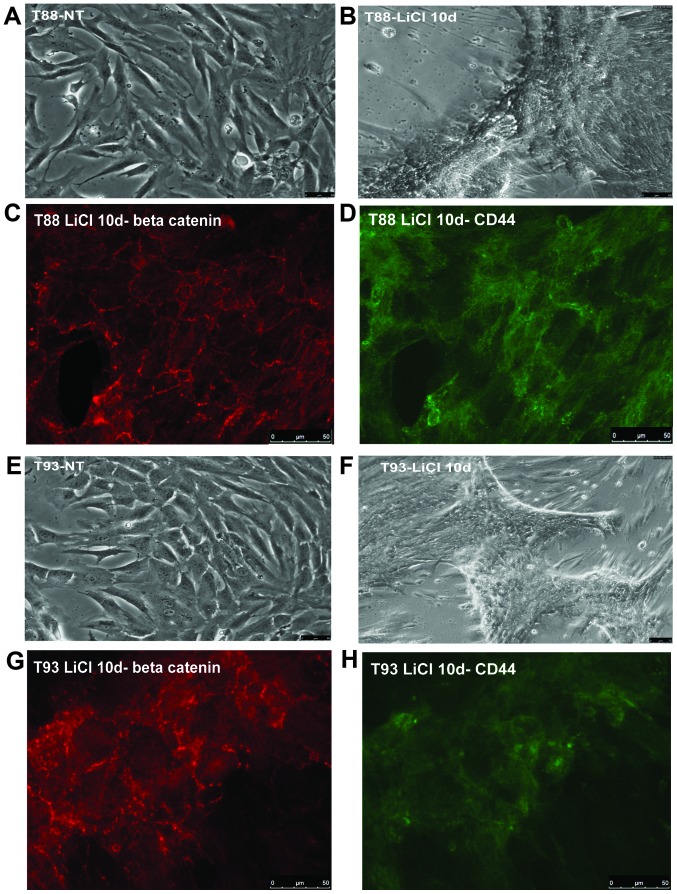
LiCl induces *in vitro* differentiation. Cell culture images in bright field of (A) T88 and (E) T93 at T_0_ and (B and F) after 10 days of LiCl incubation. Immunofluorescence assay of (C and G) β-catenin and (D and H) CD44 on differentiated cells. Magnification, ×20 (scale bar, 50 μm).

**Figure 5:**
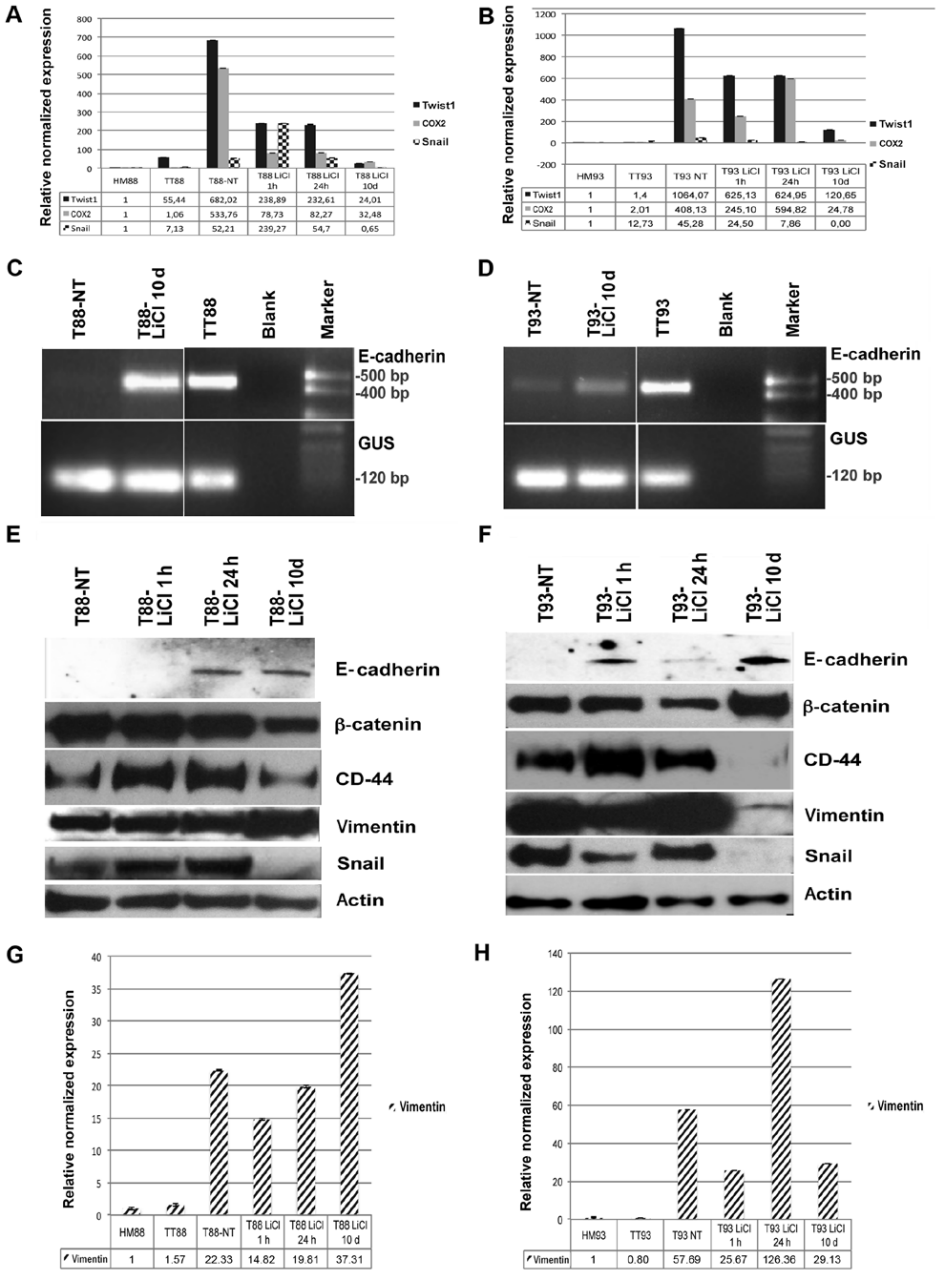
LiCl incubation of primary cancer cell cultures induces mesenchymal-to-epithelial transition (MET) *in vitro*. (A and B) Real-time RT-PCR analysis of Twist1, Snail and cyclooxygenase-2 (COX2) performed on healthy mucosa (HM), tumor tissue (TT), untreated tumor cell cultures (T), and tumor cells after 1 and 24 h as well as 10 days of LiCl incubation from patient (A) no. 88 and (B) no. 93. (C and D) RT-PCR analysis of E-cadherin performed on cDNA from HM, TT and untreated T from patient (C) no. 88 and (D) no. 93. (E and F) Western blot assay of β-catenin, E-cadherin, CD44, vimentin, Snail and actin performed on proteins extracted from untreated T, and tumor cells after 1 and 24 h as well as 10 days of LiCl incubation of (E) T88 and (F) T93 cells. (G and H) Real-time RT-PCR analysis of vimentin performed on HM, TT, untreated T, and tumor cells after 1 and 24 h as well as 10 days of LiCl incubation from patient (G) no. 88 and (H) no. 93.

Following incubation with the GSK3β inhibitor LiCl, cells differentiated, as shown in Fig. 4A, B, E and F and β-catenin, like CD44, become mainly localized at membrane level, as shown by immunofluorescence assay (Fig. 4C, D, G and H). Twist1, Snail, COX2 and CD44 expression was downregulated (Fig. 5A, B, E and F) and cells, originally negative for E-cadherin, started to express this epithelial marker both at RNA (Fig. 5C and D) and protein level (Fig. 5E and F), suggesting a mesenchymal-to-epithelial reverting transition process. The T88-untreated cells were more mesenchymal-like than the T93 cells, and did not express E-cadherin. On the other hand, the T93-untreated cells, that showed more epithelial-like morphology, had low level of E-cadherin transcription (Fig. 5C and D).

When analyzed by western blot assay, Snail and CD44 gave no signal after 10 days of LiCl incubation in T93 cells, while they strongly decreased in T88 cells (Fig. 5E and F). Interestingly, β-catenin and vimentin showed the opposite trend in response to GSK3β inhibition (Fig. 5E and F). After 10 days of LiCl incubation, expression of vimentin mRNA and protein strongly decreased in T93 cells (Fig. 5F and H) while they increased in T88 cells (Fig. 5E and G). On the other hand, in the same conditions, β-catenin was upregulated in T93 cells and downregulated in T88 cells (Fig. 5E and F).

## Discussion

We have set up a protocol to isolate and establish primary CRC cell cultures highly enriched in mesenchymal cancer cells derived by an EMT process from epithelial cells. To our knowledge, this is the first primary CRC cell culture isolated from CRC patients expressing mesenchymal and epithelial biomarkers together with high level of EMT transcription factors.

The T88 and T93 cultures were further analysed. T88 cells appeared more mesenchymal-like than T93 cells, that instead showed a more epithelial-like morphology. The cancer nature of these cells was confirmed and validated by MSI and kariotype analysis.

MSI, CpG island methylator phenotype (CIMP) and CIN play a significant role in CRC (6).

CIN occurs in ~60% of CRC and concerns different cellular phenomena chacterised by the presence of abnormal chromosome number or complement (23).

MSI consists in variations in the number of repetitive units in each microsatellite. It is caused by failure of the DNA mismatch repair system to repair errors occurring during replication. These mistakes are usually corrected by the MMR which include hMLH1, hMSH2 and hMSH6 (24). There are two well-established MSI phenotypes, namely MSI-H and MSI-low (MSI-L or MSS). We found that T88 cells showed MIN, whereas T93 cells were carriers of a 46,XX,t(20;22) (q13;q13) translocation in 95% of metaphases. Both regions on chromosome 20 and 22, involved in this translocation, are reported to be alterated in sporadic CRC (25–27). We concluded that both cell cultures were highly enriched with colon cancer cells.

Furthermore, we showed that these cells expressed mesenchymal markers, EMT-TF and epithelial markers together, therefore they have to be considered mesenchymal colon cancer cells that have undergone EMT from epithelial adenocarcinoma cells. In agreement with each morphological phenotype, T88 cells, that were more mesenchymal-like than T93 cells, did not express E-cadherin, not even at mRNA level and showed a higher increment in Twist1 mRNA expression in TT compared to T93 cells, that showed more epithelial-like morphology and had a low level of E-cadherin transcription (Fig. 5C and D).

According to literature data (28,29), these cells express some stem cell markers, such as Oct4 and Nanog and are able to grow as spheres in a conditioned medium without serum and in low adhesion condition.

We were able to induce a mesenchymal-to-epithelial reverting transition under incubation with the GSK3β inhibitor LiCl. LiCl is the most studied between GSK3β inhibitors and exerts its action through two well-known mechanisms. As a direct inhibitor, lithium is a competitive inhibitor of the Mg^2+^, which results in inhibition of the activity of this enzyme. On the other end, it also acts in an indirect manner, causing a large increase in the phosphorylation of Ser9 of GSK3β (30).

In our experience, after 10 days of LiCl incubation, cells were able to transcribe E-cadherin; Twist1 and Snail mRNA was strongly downregulated as well as CD44 and Snail protein, when investigated by western blot assay. Interestingly, COX2 mRNA was highly overexpressed in both cell cultures, compared to their matched colon mucosae and it was also highly downregulated after LiCl incubation. According to these results, literature data indicate a strong association between COX2 expression and cancer progression and metastasis (31).

As stated in the Introduction, GSK3β can act as pro-apoptotic signal negatively regulating Wnt signaling, by controlling the degradation of β-catenin. On the other hand, it positively regulates NF-κB pathway by mediating the degradation of IκB, a central inhibitor of NF-κB, so inducing an anti-apoptotic responce (32).

In colon and pancreatic cancer cells, GSK3β activates a proliferative signal by activation of NF-κB, and inactivation of GSK3β inhibits NF-κB activity (33,34).

Interestingly, *Snail*, *CD44*, *G3BP2*, and *YAP1* are targets of *Wnt5A*, a gene involved in invasion and metastasis of many cancers, that is regulated by NF-κB signaling pathway (35,36).

E-cadherin and its transcriptional repressor Snail orchestrate a functional cross-regulation between Wnt and NF-κB pathways during EMT. Expression of Snail promotes E-cadherin downregulation destroying epithelial organization and inducing mesenchymal phenotype of epithelial cells, that takes place together with upregulation of mesenchymal genes expression. E-cadherin and other cell adhesion components directly bound both β-catenin and NF-κB, sequestering them into adherens junctions and therefore preventing the transcription of target genes (37,38).

As recently described (39), we suggest that GSK3β inhibition could represent a good strategy targeting the cross-regulation between these two pathways and may be a promising direction for future cancer therapy that needs to be better elucidated.

Cell culture models obviously do not mimic the complex interaction that are found in the intestinal mucosa and a functional change in the cell cultures cannot tell us whether there will be the same effect *in vitro*. Nevertheless cell cultures are used in an attempt to make complex systems more simple by isolating certain functions and investigate them in detail. From this point of view, we speculate that our cell culture could represent an interesting model to further investigate the molecular biology of mesenchymal CRC cells, clinical relevance of EMT in human CRC and the molecular basis of pharmacological resistance and metastasis.

In conclusion, our preliminary data suggest that EMT could represent a common mechanism among very different colon cancer types and LiCl, a GSK3β inhibitor, induces MET in both cell types despite their genotype, suggesting that LiCl and GSK3β could represent, respectively, interesting drug and target for CRC therapy. However, it seems to be more efficient in T93 cells, characterized by a CIN phenotype, than in T88 cells, that showed MIN phenotype, even if the first one was a more advanced stage carcinoma. Obviously, larger population studies and further investigations will be necessary to confirm our results.

## Figures and Tables

**Table I:** Oligonucleotide sequences.

Oligonucleotides	Primers	Accession nos.
Cytokeratin 18	F: 5′-AGACTGGAGCCATTACTTC-3′	[NM_199187.1; start +441]
	R: 5′-GCTCTGTCTCATACTTGACTC-3′	[NM_199187.1; start +563]
Cytokeratin 20	F: 5′-CTGCAAATTGATAATGCTAA-3′	[XM_005277792.1; start +485]
	R: 5′-GGTCATCAAAGACCTTATTC-3′	[XM_005277792.1; start +586]
B-Raf	F: 5′-TGCTTGCTCTGATAGGAAAATGAGA-3′	[NC_018918.2; start +140387483]
	R: 5′-CTCAGCAGCATCTCAGGGCC-3′	[NC_018918.2; start +140387250]
E-cadherin	F: 5′-TCCTGGGCAGAGTGAATTTT-3′	[NM_004360.3; start +276]
	R: 5′-CCGTAGAGGCCTTTGACTG-3′	[NM_004360.3; start +381]
Snail 1	F: 5′-GCGAGCTGCAGGACTCTAAT-3′	[NM_005985.3; start +143]
	R: 5′-TCCCAGATGAGCATTGGCAG-3′	[NM_005985.3; start +269]
Twist1	F: 5′-CCTTCTCGGTCTGGAGGAT-3′	[NM_000474; start +908]
	R: 5′-TCCTTCTCTGGAAACAATGACA-3′	[NM_000474; start +1004]
Vimentin	F: 5′-TCTGGATTCACTCCCTCTGG-3′	[NM_003380; start +1694]
	R: 5′-GGTCATCGTGATGCTGAGAA-3′	[NM_003380; start +178
GUS	F: 5′-GAAAATATGTGGTTGGAGAGCTCATT-3′	[XR_242233.2; start +1695]
	R: 5′-CCGAGTGAAGATCCCCTTTTTA-3′	[XR_242233.2; start +1774]

GUS, glucuronidase.

## Abbreviations

EMT: epithelial-to-mesenchymal transition
MET: mesenchymal-to-epithelial transition
CRC: colorectal cancer
CIN: chromosomal instability
MIN: microsatellite instability
MMR: mismatch repair genes
HM: healthy mucosa
TT: tumor tissue
CIMP: CpG island methylator phenotype
