# [Corrigendum] Lithium chloride induces mesenchymal-to-epithelial reverting transition in primary colon cancer cell cultures

**DOI:** 10.3892/ijo.2025.5800

**Published:** 2025-09-09

**Authors:** Valeria Costabile, Francesca Duraturo, Paolo Delrio, Daniela Rega, Ugo Pace, Raffaella Liccardo, Giovanni Battista Rossi, Rita Genesio, Lucio Nitsch, Paola Izzo, Marina De Rosa

Int J Oncol 46: 1913-1923, 2015; DOI: 10.3892/ijo.2015.2911

Following the publication of the above article, an interested reader drew the authors' attention to the fact that the CTK18 panel in Fig. 2E on p. 1917, showing the results of RT-PCR analysis of cytokeratin 18 from patient no. 88, appeared to be very similar to the CTK18 panel in Fig. 2F (showing the results from patient no. 93).

After having re-examined their original data, which were also presented to the Editorial Office, and considering that the observed experiment is an end-point RT-PCR performed more than ten years ago, the authors cannot definitively rule out the possibility that Fig. 2E was inadvertently misassembled. Therefore, given the high similarity of the two images, it was decided to publish a revised version of Fig. 2, which now shows data from a different replicate of the experiment for the CTK18 panel in Fig. 2E, shown on the next page. Note that this revision did not affect the overall conclusions reported in the study. The authors are grateful to the Editor of *International Journal of Oncology* for allowing them this opportunity to publish a Corrigendum, and all the authors agree with its publication. Furthermore, the authors apologize to the readership for any inconvenience caused.

## Figures and Tables

**Figure 5:**
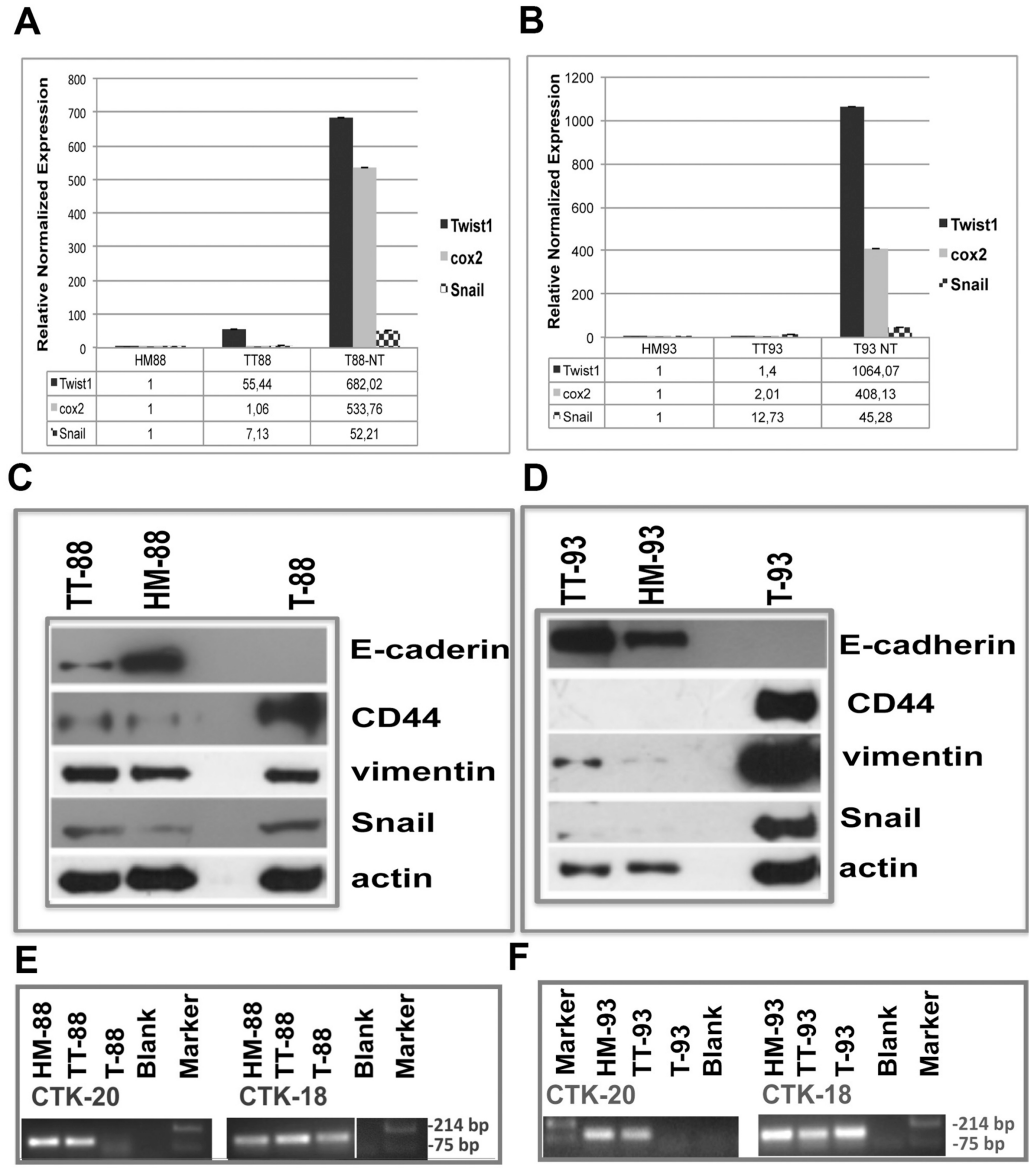
LiCl incubation of primary cancer cell cultures induces mesenchymal-to-epithelial transition (MET) *in vitro*. (A and B) Real-time RT-PCR analysis of Twist1, Snail and cyclooxygenase-2 (COX2) performed on healthy mucosa (HM), tumor tissue (TT), untreated tumor cell cultures (T), and tumor cells after 1 and 24 h, as well as 10 days of LiCl incubation from patient (A) no. 88 and (B) no. 93. (C and D) RT-PCR analysis of E-cadherin performed on cDNA from HM, TT and untreated T from patient (C) no. 88 and (D) no. 93. (E and F) Western blot assay of β-catenin, E-cadherin, CD44, vimentin, Snail and actin performed on proteins

